# Overcoming Barriers to Successful Climate and Health Adaptation Practice: Notes from the Field

**DOI:** 10.3390/ijerph19127169

**Published:** 2022-06-11

**Authors:** Evan Mallen, Heather A. Joseph, Megan McLaughlin, Dorette Quintana English, Carmen Olmedo, Matt Roach, Carmen Tirdea, Jason Vargo, Matt Wolff, Emily York

**Affiliations:** 1 Climate and Health Program, US Centers for Disease Control and Prevention, Atlanta, GA 30333, USA; hbj7@cdc.gov (H.A.J.); qxd4@cdc.gov (M.M.); 2 Climate Change & Health Equity Section, Office of Health Equity, California Department of Public Health, Sacramento, CA 95814, USA; dqe1@att.net (D.Q.E.); jason.vargo@sf.frb.org (J.V.); 3 Population Health Division, San Francisco Department of Public Health, San Francisco, CA 94102, USA; carmen.olmedo@sfdph.org (C.O.); matt.wolff@sfdph.org (M.W.); 4 Climate and Health Program, Office of Environmental Health, Arizona Department of Health Services, Phoenix, AZ 85007, USA; matthew.roach@azdhs.gov (M.R.); carmen.tirdea@azdhs.gov (C.T.); 5 Oregon Climate and Health Program, Public Health Division, Oregon Health Authority, Portland, OR 97232, USA; emily.a.york@dhsoha.state.or.us

**Keywords:** adaptive capacity, climate change adaptation, resilience, evaluation

## Abstract

State and local public health agencies are at the forefront of planning and responding to the health challenges of climate hazards but face substantial barriers to effective climate and health adaptation amidst concurrent environmental and public health crises. To ensure successful adaptation, it is necessary to understand and overcome these barriers. The U.S. Centers for Disease Control and Prevention Climate-Ready States and Cities Initiative (CRSCI) provides funding to state and local health departments to anticipate and respond to health impacts from climate change using the Building Resilience Against Climate Effects (BRACE) framework. This paper explores the barriers to and enablers of successful adaptation projects among BRACE West CRSCI grantees, including Arizona, California, Oregon, and the city and county of San Francisco. The barriers included competing demands such as the COVID-19 pandemic, dependence on partners with similar challenges, staff and leadership turnover, uncertain and complex impacts on at-risk populations, and inadequate resources. The enablers included effective partnerships, leadership support, dedicated and skilled internal staff, and policy windows enabling institutional change and reprioritization. These findings highlight effective strategies in the field that state and local health departments may use to anticipate potential barriers and establish their work in an environment conducive to successful adaptation.

## 1. Introduction

State and local public health agencies are at the forefront of planning and responding to the health challenges of climate hazards such as heat waves, wildfires, storms, flooding, drought, adverse air-quality events, and sea level rise [1]. Adaptation to climate change often refers to intentional planned actions by individuals, social groups, or institutions to avoid, prepare for, or respond to the detrimental impacts of observed or anticipated climate change [2,3,4]. Adaptation actions can include policy, regulation, single strategy projects, or multi-component programs [5] and are always embedded in a context of environmental, demographic, cultural, and economic change [6]. In part due to the emerging and dynamic characteristics of climate change and society’s response, adaptation practitioners have faced interrelated challenges to planning and implementation that can stop, delay, or divert progress in achieving climate resiliency [7].

This is also true in the context of the health sector. The USA devotes less than 3% of health spending toward public health and 97% toward health care [8], and since 2010, spending for public health departments has dropped by 16% per capita [9]. The USA faces an estimated $4.5 billion shortfall in funding to provide a minimum standard of foundational public health capabilities [10]. Prior to the COVID-19 pandemic, the public health workforce had lost at least 38,000 state and local jobs since the 2008 recession [11]. Assessments of public health competency by agency workers and their supervisors have identified gaps in mastery needed for effective practice [12]. These gaps have been documented in key competencies, such as using evidence in decision making [13].

These challenges affect the nation’s public health capacity across many critical services, such as infectious and chronic disease, as well as disaster preparedness. There are additional challenges for those engaging in climate and health adaptation. Many have called for the field to prioritize research that clarifies the current and potential future health impacts of climate change, such as scenario-based projections of impacts, maximizing the health benefits of climate mitigation investments, and assessing the cost-effectiveness of potential adaptation options [14]. However, at the local and state level, health departments have reported limited subject matter expertise to research current and future health impacts from climate change or to implement and evaluate interventions to reduce those impacts. One study found that only 5.1% of health departments “strongly agree” that they had “expertise to assess the potential public health impacts associated with climate change” [15]. A survey by the National Association of County and City Health Officials (NACCHO) found that 76% of county health directors believed their health departments lacked the expertise to assess potential climate impacts, 80% said they lacked the expertise to create effective plans to protect residents from the health impacts of climate change, and 87% indicated that their health departments did not have sufficient resources to effectively protect local residents from the health impacts of climate change [16]. Other barriers include institutional limitations such as lack of leadership or supportive political environments, attitudes among the public, and lack of information or resources [7,17,18,19,20,21]. Recent public health, environmental, and social crises have significantly affected the abilities of state and local health departments to implement their essential services.

Unless significant changes are made to U.S. public health system funding and organization, this challenging landscape is likely to persist or worsen as the impacts of climate change intensify and further stress local and state governments. However, Ekstrom et al. [7] argue that barriers may be overcome with “concerted effort, creative management, change of thinking, prioritization, and related shifts in resources, land uses, institutions, etc.” By gaining the necessary skills, learning, and adapting through ongoing work, health departments can build institutional capacity allowing them to overcome barriers more easily in the future [22,23]. Identifying and leveraging the conditions that facilitate climate adaptation can help health agencies build their capacity to address climate impacts [24].

Emerging crises may also create “policy windows” that provide an opportunity to achieve public health goals, including climate and health adaptation [25,26]. Policy windows are important agenda-setting opportunities [27]. They are “exceptional, fleeting periods of time when there is a greater likelihood of initiating policy change than usual” [28]. This disruption of the normal status quo policy environment can happen when three independent streams converge—the political stream (public opinion and the state of politics), the policy stream (potential solutions to the problem), and the problem stream (attributes and trends of the problem, whether it has gained attention through a focusing event, and whether potential solutions in the policy stream can solve or mitigate the problem) [28]. These policy windows may allow health departments to acquire or shift resources and institutional focus to address public health needs stemming from climate change. When these streams converge, it may help to reframe climate change as a public health problem and elevate it as a high-priority issue. This has been found to be especially true when there is ambiguity in the policy process characterized by unclear goals, multiple and diverse actors, and unclear roles and responsibilities in addressing the problem [29]. With complex and wide-ranging impacts from climate change, it is worth examining if such windows can be anticipated and strategically used by practitioners and their partners to establish policies and resources to further their missions of protecting at-risk populations against the impacts of a changing climate.

The Climate Ready States and Cities Initiative (CRSCI) was developed in 2009 by the U.S. Centers for Disease Control and Prevention (CDC) to build state and local health department capacity to address the health effects of climate change. The initiative’s flagship Cooperative Agreements (CDC-RFA-EH12-1202; CDC-RFA-EH13-1305; CDC-RFA-EH16-1602) have funded 18 public health jurisdictions to build capacity and implement the Building Resilience Against Climate Effects (BRACE) framework [5,30]. BRACE is a sequential framework that guides public health agencies to anticipate the impacts, assess the vulnerabilities, project the disease burden, assess public health interventions, develop and implement a climate and health adaptation plan, and evaluate engagement and impact.

Stakeholder engagement and adaptive management practice are two central principles to the conceptualization and implementation of the BRACE framework [31]. Adaptive management is a well-established learning-based approach to the design and implementation of interventions in a complex dynamic system where there is incomplete understanding. This requires regular revision of strategies via stakeholder learning that stems from interactions with the system and each other [32,33,34]. Such revision, or novelty, can enhance system resilience and build adaptive capacity [35,36]. A key attribute of this approach is the use of conceptual models and gathering information and insights periodically through iterative evaluation and monitoring and the ability to change course based on changing or unforeseen conditions [37]. This foundational principle of BRACE underscores the need to regularly assess the factors contributing to, as well as undermining, outcomes in terms of implementing strategies that are building resilience to climate change. Unfortunately, there has been little systematic evaluation to establish the effectiveness of adaptive management as an overall planning strategy. More recently, some have called for new approaches for climate adaptation planning that address some of the challenges observed with adaptive management in practice [38,39,40,41]. Anticipatory governance has been proposed as a promising update to adaptive management and has been defined as “a system of institutions, rules and norms that provide a way to use foresight for the purpose of reducing risk, and to increase capacity to respond to events at early rather than later stages of their development” [42,43,44]. Another challenge specific to adaptive management in public health is the field’s emphasis on using evidence-based interventions to achieve public health goals [45,46,47,48]. The generation of such evidence is a time consuming and resource intensive process. It remains unclear how the emphasis on implementing evidence-based interventions will coalesce with the urgent, front-line, and context specific need for often under-resourced local agencies to take immediate action to protect health in the context of rapid climate change [49,50,51].

A better understanding of the barriers and enablers to climate and health adaptation amid disruptions will help practitioners better anticipate and overcome such barriers, enhance adaptive capacity, and enhance local resilience. In this paper, we explore what CRSCI grant recipients in the Western USA (BRACE West) have learned through implementing eleven climate and health adaptation projects in their local context. In the sections that follow, this paper will cover five main objectives: (1) describe successful climate and health adaptation initiatives among four BRACE West grant recipients; (2) describe how local implementers define building capacity; (3) characterize the barriers and enablers encountered in their implementation; (4) describe whether and how barriers were overcome to enhance adaptive capacity; and (5) propose best practices for health departments engaging in climate and health adaptation efforts.

## 2. Materials and Methods

We used a case study approach considering eleven projects from four CRSCI grantees located in the Western USA. The four recipients were the Arizona Department of Health Services, the California Department of Public Health, the Oregon Health Authority, and the San Francisco Department of Public Health. Project teams (consisting of the project members who are co-authors on this paper) shared descriptions of two to four successful adaptation interventions or actions (i.e., cases) for inclusion. Success was locally defined, and in all cases, the teams determined that the action was able to meet the initial objectives.

To synthesize the results, the project teams began by sharing written success stories previously prepared for CRSCI annual grant reporting requirements. To further standardize the information, project teams collaboratively established key elements to include in written narrative descriptions of projects. The project teams prepared and shared these more standardized written descriptions and then collaboratively identified common themes. Each team distilled key elements of their projects into tables (e.g., stakeholders, barriers, and enablers).

Project teams participated in semi-structured discussions to further elucidate emergent themes from these materials. In the first round of discussion, leads discussed barriers encountered in each of their cases, and to what extent they were able to overcome each barrier. The term “barrier” was defined to be “a process, action, or condition that delays the timeliness or quality of a project deliverable.” Each team also discussed factors that helped facilitate their work, which are referred to here as “enablers.” Enablers were defined to be “an existing resource, skill set, personnel, or other enabling factor present that helped the team conduct the project smoothly.”

Staff also discussed major disruptions that inhibited progress or served as policy windows to spur change. Each team also reviewed a compiled list of barriers and enablers that emerged from their narrative descriptions to confirm whether each of the barriers and enablers in turn applied to their projects. Finally, for a broader context on how these experiences influenced their adaptation efforts, they discussed how these experiences influenced how they operate now or will in the future, and how they can continue to build capacity to overcome future disruptions.

Notes generated through these discussions were entered into a qualitative analysis software package, Dedoose (v8.3.47b, SocioCultural Research Consultants, Manhattan Beach, CA, USA), and standard qualitative coding was performed by two CDC co-authors to document, compare, and contrast key themes from across the eleven cases. Notes were coded by topic across all four grantee teams to inductively determine emergent themes across the grantee responses. The CDC co-authors synthesized themes through multiple rounds of internal discussion to organize into common themes for barriers and enablers identified by the grantee teams.

### Case Descriptions

The project team leads provided eleven cases in total, representing successful examples of state and local climate and health adaptation initiatives. Each case is a project representing actions, interventions, or agency adaptations intended to reduce the negative health impacts of climate change funded through the Climate Ready States and Cities Initiative (CRSCI) Cooperative Agreement. Each grantee team consists of state or local health department staff, including internal or external collaborators. These cases are summarized in Table 1.

## 3. Results

### 3.1. Practitioner Conceptualization of Capacity Building and Associated Challenges

In defining capacity in the field of climate change and health, recipients identified two overlapping essential elements: professional climate literacy and collaboration with partners. Several project teams described climate literacy as the knowledge and understanding of climate and health topics, ability to summarize climate adaptation, and engage with other practitioners about the technical aspects of the field. A common theme was that building capacity within health departments required internal personnel with specific training, knowledge, and skill sets. To achieve this, health departments can hire dedicated staff, fellows, and interns, and create workforce development plans. Hiring climate and health staff with expertise on the health benefits of adaptation across departments external to public health such as transportation, natural resources, and behavioral sciences was also considered beneficial. It was shared that building capacity helps to institutionalize climate change in the public health system such that if any staff leave, the program will not collapse as a result.

Project teams consistently shared that capacity could also be augmented through partnering with organizations and agencies to leverage external resources and reach common goals. The San Francisco team observed that emergency preparedness and public health are often planned according to short timelines and often reactive as a result, making these planning processes susceptible to disruption. For the Arizona team, building capacity also meant developing thoughtful strategic plans informed by both the literature and stakeholder input. Such planning helps address both short-term and long-term strategies such as the long-term green infrastructure strategy outlined in Maricopa’s strategic plan [52].

Three of the four project teams also described challenges unique to building capacity for climate and health adaptation. The California and Oregon teams articulated that the scope and complexity are uniquely challenging for climate adaptation. There are many interacting hazards, sectors, and vulnerabilities that are constantly shifting. These variables make it difficult to quantify climate change as a static health threat and to understand and predict its likely impacts on specific communities. As the impacts of climate change are unfolding so rapidly, climate and health adaptation requires continuous analysis, learning, and skill development. The California and Arizona project teams shared that staffing is uniquely challenging for climate work. Generally public health staff are highly specialized within their subdisciplines, which rarely include climate change-specific training. As a result, internal climate initiatives do not have operational infrastructure or seasoned staff, and it can be difficult to identify personnel with adaptable skill sets and expertise that are transferable to climate work. It is necessary to look at subject matter experts outside the agency to help aid climate initiatives such as university researchers. Cost is also a major factor, as teams reported many constraints on public resources, and efforts to better understand climate impacts or projections through universities come with high overhead costs. One team also commented on the political nature of climate change, as the will and means to pursue climate adaptation may shift with political leanings of a jurisdiction or current administration. The San Francisco and Oregon teams noted that climate change is more regional in scope than other public health work, with impacts across city, county, tribe, state, and international boundaries; so, climate work requires collaboration across scales and jurisdictions that may not currently jointly plan for and respond to public health-related aspects of climate-related hazards.

### 3.2. Barriers Encountered While Implementing Climate and Health Adaptation

All eleven projects faced barriers to achieving success, impacting the timeliness or effectiveness of the final products or outcomes. Five major categories of barriers emerged from the cases presented: (1) competing demands, (2) dependence on partners with similar challenges, (3) staff and leadership turnover, (4) scientific uncertainty and complexity, and (5) inadequate resources. The barriers and enablers are summarized by project in Table 2.

#### 3.2.1. Competing Demands

Competing demands on time and resources was the most significant barrier to progress for all four teams, impacting ten of the eleven projects. No disruption was as significant as COVID-19, which emerged as a pronounced theme in nearly all eleven projects. Many of the issues raised were attributable to the COVID-19 pandemic taking priority over climate and health adaptation work in favor of a high-priority near-term emergency response. The pandemic was named as directly or indirectly responsible for most of the barriers involving competing demands, minimal time to devote to climate, and staff turnover, all reducing overall staff capacity to address climate and health work. The pandemic response also caused logistical problems for coordination between staff and external stakeholders, who were also consumed with COVID-19 related challenges. Some activities were delayed, preventing important climate messaging from reaching intended audiences in a timely manner. In Arizona, some partners withdrew new project proposals to focus on the COVID-19 response, which resulted in a one-year setback on their projects. When schools went to virtual learning in 2020, the Arizona team had to wait until the following year when schools returned to in-person teaching to pilot their school heat policy document while experiencing record-breaking heat in 2020. Similarly, the Climate Change Coordination Committee in San Francisco was delayed up to seven months due to COVID-19 demands on staff time, limiting their capacity to engage stakeholders, and causing a reduction in scope and reach.

Other competing priorities also disrupted climate work in San Francisco, such as concurrent crises in drug overdosing-related health challenges, behavioral health, and housing affordability. The California project team reported that political disruptions often impacted the reach and scope of statewide initiatives. Some county leaders in both urban and rural areas imposed restrictions that made local health officials reluctant to visibly engage in climate and health work, rendering these departments unable to plan for climate change threats openly, though they engaged in capacity building work and accessed state resources. On the environmental hazard side, events such as wildfires [53] also demanded immediate attention, which further reduced staffing and resources devoted to long-term adaptation planning and institutionalizing climate work. One project lead noted that addressing the root causes in climate and health adaptation is under-resourced or neglected unless there is a crisis.

Before the pandemic, teams also experienced challenges related to staff time. One team noted that in terms of collaborating with stakeholders, the topic of climate change necessitates more time than other public health issues given the novelty and complexity of the issues. Another noted that the tools used in climate communication require constant evaluation and updating, and that time constraints limited robust knowledge dissemination, diverse stakeholder input, and iterative development and improvement.

#### 3.2.2. Managing Diverse Stakeholder Needs among Partners Facing Similar Challenges

Dependence on external partners with similar time and resource-related challenges was also found to be a significant barrier across all four teams in ten of the eleven projects. These partnerships included stakeholders from external organizations and from the general public. Stakeholder engagement was an essential component of many of the projects to gather information, leverage external skill sets and knowledge, and to help tailor the interventions. However, varying stakeholder needs and perspectives made designing and scoping the interventions difficult. For California, it was difficult to meet the demands of multiple stakeholders with varying levels of training and engagement with a single tool. Oregon found it difficult to coordinate climate work across twenty agencies each with differing needs while providing best practices relevant to all stakeholders. San Francisco had difficulty developing guidance to prepare hospitals for high heat and poor air quality days when each hospital staff role (i.e., administrator, clinician, facilities) requires a unique strategy. Each of the teams noted that their projects would have been improved with more time to better engage and learn from the diverse stakeholders early on and throughout the process, if time was not so limited. Gathering stakeholder input from the beginning would increase the likelihood that the work was best meeting their diverse needs, and that tools or resources were accessible and user-friendly. Limited coordination with partners and stakeholders due to lack of time or internal capacity was also a major barrier to success across all four teams. In some cases, it was difficult to convene stakeholders in one place, in part due to the COVID-19 pandemic. In San Francisco, this resulted in a need for one-on-one interviews, which did not allow for discussion of multiple perspectives at once. In Oregon, high partner turnover made coordination more difficult.

#### 3.2.3. Staff and Leadership Turnover

Staff and leadership turnover were significant barriers for all four teams, impacting seven of the eleven projects. New staff with varying skill sets required constant training, which took time away from already time-limited projects. In some instances, new leadership brought with them new priorities that required additional staff training. Intermittent leadership buy-in also resulted in uncoordinated project prioritization and disjointed responsibilities among the staff. Staff and leadership turnover among partners external to the health department also caused delays or reduced quality of work due to shifts in available skill sets and expertise. Such staff turnover was mostly attributable to the COVID-19 pandemic redirecting time and resources, but also included term-limited fellowship appointments expiring and lateral movement to other departments due to COVID-19 and climate burnout.

#### 3.2.4. Uncertainty and Complexity

The uncertainty and complexity of climate and health adaptation were major barriers to three of the four teams across seven of the eleven projects. Limitations in locally relevant data and literature, as well as the complex socioecological interactions involved in climate change made it difficult to identify disproportionately impacted populations to prioritize and determine the most effective response. In California, lack of timely syndromic surveillance data in general and micro-scale data among transient populations or those difficult to track, such as unhoused populations, migrant, or undocumented farm workers, or small Native American and rural populations added to the uncertainties in determining how best to intervene. In San Francisco and Arizona, lack of adequate heat exposure data, such as readily available temperature data to link to heat-related emergency department visits in school-age children, hindered efforts to design targeted interventions.

#### 3.2.5. Inadequate Resources

Inadequate funding was a barrier for all four teams, impacting five of the eleven projects. Funding specifically devoted to the climate portfolio was essential to support staff time for the effort and to prevent it from being diverted toward other work, such as the COVID-19 response. Funding restrictions regarding onerous fiscal and contracting procedures and protracted hiring timelines also caused issues as certain funds were unable to be spent on relevant and necessary climate and health adaptation. The California project lead noted that the resources available did not match the amount of work required regarding the magnitude of the problem of climate change and the number of people affected. For example, the CCHViz project created useful indicators for climate vulnerability but did not have an outreach budget to fully engage stakeholders. Dissemination may have been more impactful with funding for training webinars or outreach at local sites and events to create more community connections while promoting the tool’s use across the state. More comprehensive engagement would have promoted further adoption by more stakeholders who could further apply the data and information in the tool. Similarly, the CalBRACE Adaptation Toolkit may be more effective with additional staff time dedicated to deeper tool use evaluation and metrics, such as quantifying clicks or downloads on specific elements on the website or hosting focus groups to better understand user behavior to guide updates. Such iterative evaluation could improve the product over time by adapting to better meet stakeholder emerging needs. Without direct funding on the local level for dedicated climate change staff, there was limited bandwidth for local health department staff, impacting the project’s reach and impact in local and regional planning actions.

### 3.3. Enablers That Helped Climate and Health Adaptation Succeed

As expected, the case studies of successful projects had enabling factors that facilitated successful climate and health adaptations and overcoming or limiting the barriers mentioned above. Four categories of enablers emerged: (1) effective partnerships, (2) leadership support, (3) dedicated and skilled internal staff, and (4) policy windows enabling institutional change and reprioritization.

#### 3.3.1. Effective Partnerships

The first set of enablers regards successful partnerships including CDC funding and support, partner skill sets, and leveraging ongoing work. These aspects enabled all eleven projects across the four teams. A common theme among the barriers above was related to limited time and staff devoted to climate and health adaptation. Funding and technical support from the CDC CRSCI Cooperative Agreement enabled project staff on all four teams to dedicate staff time explicitly to climate work, and all four project teams noted that much of the work would not have been possible without this support. In addition to staff time, the grant funding gave the teams additional flexibility to build capacity where possible, such as through hiring new fellows or consultants to bring skills and expertise. Secured funding also prevented some staff from being pulled away for the COVID-19 response and kept the climate work moving forward. The BRACE framework helped facilitate some projects by providing a sequenced structure for their work and reduced duplication of efforts. The CDC also provided technical assistance and communities of practice on evaluation, which helped the recipients connect the climate work to health outcomes in new ways.

Funding for stakeholders was also an important feature of successful partnerships for climate and health adaptation work. In San Francisco, leaning on other well-funded departments was an effective way to keep climate work moving forward and to secure a seat at the table with well-resourced organizations without the same barriers resulting from COVID-19. California leveraged the significant resources of state agency partners in natural resources, energy, land use, and transportation agencies to include the CCHVI indicators and CCHViz tool, as ways for these agencies to prioritize grant and policy resources to communities facing combined climate and health exposures and vulnerabilities. California coordinated with the California Climate Action Team convenings and efforts to promote utilization of the CCHVIs in state agencies’ adaptation and mitigation actions. In Arizona, BRACE funding allowed partnerships with Arizona State University to help facilitate state heat preparedness meetings, which would not have been possible otherwise [54]. In Oregon, funding enabled the team to hire consultants to help facilitate climate equity trainings for state agency partners.

Building capacity through leveraging partner skill sets and initiatives were also major enablers to all four teams. Arizona and California developed productive relationships with university partners, bringing climate science, health, data collection, vulnerability assessment, and evaluation expertise. California leveraged external fellowships through CivicSpark fellowships to take on projects with local health departments, universities, and civic organizations. Partners utilized the final products of both California projects, and broader access to the products was enhanced through such partnerships. Several teams utilized state resources such as state climate networks for existing and projected hazard data or state weather services. Other partners were recruited for specific skill sets, such as creating bilingual ESRI story maps in Arizona or community health workers trained with skills in creating simple language and two-way communication in the Oregon listening sessions [55]. San Francisco benefited from best practices knowledge outside of the climate domain, such as from emergency preparedness coordinators, clinicians, and healthcare facility managers. Furthermore, San Francisco viewed successful partnerships as a major outcome of their projects, as these partnerships enabled other climate plans and strategies for health and air quality. In Arizona, recipients utilized the National Weather Service and media connections to promote their work and that of their partners at Arizona State University.

#### 3.3.2. Leadership Support

Leadership buy-in was also a major enabler for all eleven projects. Having consistent amenable leadership enabled project staff to devote time toward climate and health adaptation, which would not have been possible otherwise. In California, leadership was committed to the project’s tools aligned with the CDC BRACE framework, enabling use of health and equity data and resources for adaptation planning and actions by state agencies, counties, and municipalities. In San Francisco and Oregon, leadership elevated climate adaptation as a department priority, demonstrating that time needed to be invested to generate capacity and motivate staff. Leadership that was invested and embedded in the process also enabled the work by enhancing messaging and resource sharing among partners. In San Francisco, climate and health adaptation was structured around initiatives to which the mayor had already committed, with a broader emphasis on health and air quality than through the lens of infrastructure alone.

#### 3.3.3. Dedicated and Skilled Internal Staff

Having dedicated internal staff with the necessary skill sets to engage in climate adaptation was an enabler for ten of the eleven projects across all four teams. Much of the work in these projects would not have been possible without dedicated staff time allowing for more robust conversations and collaboration with internal and external partners on complex issues. In California, dedicated and skilled climate and health staff guided local health departments and regional collaboratives for adaptation, as well as others focused on public health and health equity, and applied their knowledge advancing policy recommendations and applying selected CCHVIs to visualize and address complex emerging challenges such as COVID-19, systemic racism, and other factors that interact with climate exposures. In Arizona, local knowledgeable staff were able to smoothly conduct literature reviews, engage stakeholders, create graphics, conduct social media outreach, post tracking analytics, and draft and publish findings.

#### 3.3.4. Utilization of Policy Windows

Changes in the social, political, and environmental landscape provided three project teams with policy windows, or opportunities to redirect focus and priority toward climate and health adaptation work. In Arizona, the team leveraged the record-breaking heat and adverse outcomes to obtain increased prioritization, visibility, and approval for projects during COVID-19. The unprecedented chronic heat waves that Arizona experienced in 2020 provided an environmental landscape for a policy window. Reflecting the problem stream, Phoenix experienced a record breaking 48 days of Heat Warnings and 145 days over 100 °F. By comparison, from 1981 to 2010, the annual average was 110 days over 100 °F. Arizona recorded 522 heat-related deaths in 2020; the previous annual average between 2010 and 2019 was 191 [56]. Indicative of the political stream, and despite the COVID-19 crisis, this event raised awareness among the public and public health authorities to the dangers of extreme heat. This specifically helped to obtain the approvals needed to implement a heat intervention. In the policy stream, the team was approved to conduct a heat awareness campaign that included COVID-19. For example, messaging was approved that focused on protections against heat-related illness for healthcare workers performing outdoor testing for COVID-19 in the summer or reminding people to use their vehicle’s air-conditioning to lower the risk of heat-related illnesses while participating in a drive thru COVID-19 testing site.

In California, the problem stream entailed climate change and environmental hazards intensifying, reflected through increasingly costly, frequent, and disruptive events that captured the attention of decision makers, the media, and the public. Major climate occurrences included record-breaking wildfires and smoke events such as the 2018 Camp Fire with 88 deaths and the 2020–2021 wildfire events [57,58,59]. Some of these events were intensified by coinciding heat waves and public safety power shut offs to prevent wildfire ignition during windstorms. The disproportionate impacts of these events on people of color, older adults, people with disability or chronic conditions, people lacking transportation, and the unhoused highlighted structural drivers of health. The political stream galvanized public opinion about the need for the government to respond to climate threats and to bring social justice to the center of the process for people without adequate financial resources in their interaction with climate intensified events. In the policy stream, California had adopted legislation to develop cap and trade policies, legislation more favorable to adaptation, health, and other mechanisms that prioritize research, nature-based solutions to secure food systems, water and air quality, community vulnerability, and resilience to climate impacts [60]. These streams converged as an agenda-setting opportunity to elevate climate and health adaptation and emphasize the work of the California team. Spurred by the severity of the health impacts of recent heat waves and wildfires, the 2021 state budget provided for the Governor’s Office of Planning and Research to develop a Vulnerable Communities Platform that holistically identifies the communities most vulnerable to climate change, informed by the CCHVIs and CCHVIz platform as model frameworks, and with input from CDPH climate staff [61]. The 2022 budget proposes the first significant funding for climate and health activities. First is a proposal to include CDPH serving as administrator for the state’s 320 emergency departments to report syndromic surveillance data regarding patient visits for climate change-related conditions such as heat-related illness to a federal syndromic surveillance program within 24 h of the visit. Second, the budget provides one-time three-year funding for local health departments, tribes, and community-based organizations to collaborate to develop regional climate and health resilience plans based on the five health officer regions in California.

In Oregon, the problem stream entailed worsening trends in racial, economic, and health disparities due to increasing inequality. The COVID-19 pandemic and 2020 wildfire events highlighted extreme disparities, providing major focusing events for adequate public health response. In the political stream, increasingly supportive public opinion for racial equity and climate resilience provided an opportunity for legislators and elected officials to prioritize these in legislation and policy. In the policy stream, the public health agency was poised to respond to climate and social justice crises with solutions that address both issues simultaneously by including climate action within the state’s new public health investments. The Oregon team was well positioned to use the convergence of these streams as a policy window to prioritize investments in climate equity through its efforts to highlight the needs of local and tribal health departments and community-based organizations. They were also able to leverage its cross-sector work to develop the State of Oregon Climate Equity Blueprint [62], which now had broadened interest and an expanded audience. The Oregon Health Authority also hired climate equity consultants to convene partner state agencies and used CDC funding to build interagency relationships and integrate health equity strategies into the State Agency Climate Change Adaptation Framework [63] that guides climate investments across the state enterprise.

### 3.4. Climate and Health Adaptation Amid Disruptions

The concurrent crises in climate change and public health have caused major disruptions in adaptation efforts, resulting in significant barriers to progress. The barriers identified in these case studies have confirmed findings from previous studies, such as those stemming from competing demands or staff and resource limitations [17,18,19,20,21,22]. Uncertainty in climate impacts was also identified in a majority of the projects, confirming challenges found by Stults et al. [37]. Each of these barriers impacted effectiveness or timeliness of the products and outcomes of each project. Emerging crises may further disrupt standard health systems practice with new complexities and uncertainties. The 2021 Lancet Countdown Report [64] characterizes climate change as a “threat multiplier,” compounding the diverse impacts of environmental hazards resulting in excess stress on health system capacity. For example, there is increasing evidence that exposure to wildfire smoke is associated with higher rates of contracting and dying of COVID-19 [65,66,67]. On the health system side, COVID-19 related shortages in staff, equipment, and supply chains reduce system capacity to provide care for other health threats, including from increasingly likely environmental hazards. Among the eleven projects, no disruption was as great as the COVID-19 pandemic for the projects discussed here. The pandemic compounded the already difficult constraints on the public health system and added to the challenge of building capacity in the climate and health field given the complex and variable nature of the problem.

Some disruptions were viewed positively as catalysts for change. In California and Oregon, the problem, politics, and policy streams converged to facilitate significant advances in statewide climate adaptation initiatives with a focus on health equity. Major disruptions are inevitable and are expected to increase in frequency and intensity in a changing climate. State and local health departments can learn to adapt to these scenarios and use them productively for system change to enhance resilience [35]. To manage the uncertainty and complexity of such disruptions, practitioners may integrate the principles of adaptive management into their near and short-term planning [32,33,34]. Adaptive management requires practitioners to systematically assess the current state of the environment in response to ongoing changes, characterized as social, economic, or environmental indicators tracked over time. They must then generate a range of alternative objectives contingent on the scope and magnitude of these changes and design policies to achieve them. Adaptive capacity indicators for organizations include readiness to leverage governance, operational mechanisms, and policy in service to climate and health. This includes access to and understanding of climate projections, population assets and vulnerabilities, governance systems, and other aspects to implement the steps of the BRACE framework. Similarly, a framework of anticipatory governance uses indicators of change as “sign-posts,” which, when exceeding a predetermined threshold, become “trigger points” to enact policies to respond [44]. McKay et al. [68] have identified a Quality Governance Framework and Diagnostic Capacity Tool to improve the quality of socioecological systems (SES) governance. Their framework theorizes that governance that includes capacities such delegation and coordination of authority across domains and scales, participatory and network-based decision making, systems thinking, and structured deliberation. It also highlights the importance of facilitating communication and interaction between and among those who design, choose, and endure the policies enacted [68].

Some efforts to understand how adaptive management works in the field have uncovered notable challenges [38,39,40,41]. Stults et al. [37] have identified 13 distinct types of climate change uncertainty that affect planning. They also reviewed prominent planning techniques and noted that not all attempt to actively engage uncertainty. Their review of U.S. climate adaptation plans found that no communities used scenario planning or robust planning strategies despite encouragement from the peer reviewed literature. Stults et al. (2020) recommend that planners better understand the sources of uncertainty and the techniques available to help reduce that uncertainty. Notably, they do not consider the influence of uncertainty about major external disruptive events (e.g., global pandemics) that can also inhibit progress, as highlighted in this paper’s case studies.

Effective environmental management will require a mutual understanding between stakeholders and decision makers through enhanced engagement, outreach, and buy-in to co-develop policies that better serve stakeholders’ diverse needs. Anticipating how the problem, political, and policy streams converge can encourage more proactive response to these emerging policy windows as opportunities for change. Kotter [69] argued that successful institutional change requires establishing a sense of urgency, creating a new vision, removing obstacles to this vision, and institutionalizing the change. Disruptions such as the rising threat of environmental hazards described here may provide the sense of urgency necessary for state and local health departments to institutionalize the changes needed to enhance resilience to climate change in the future.

The enablers identified in this analysis were common to almost all the eleven projects, suggesting that these may be prerequisite conditions for successful climate and health adaptation. Dedicated staff and funding, both internal and external to the program, help to institutionalize the work and minimize impacts in the event of major disruptions. Cross-sector partnerships help to build climate literacy and utilize the diversity of skills across disciplines necessary for adequate interpretation and response to climate change. Some disruptions also served as enablers, resulting in policy windows for several of the project teams, providing opportunities to prioritize climate work with a focus on social and environmental justice.

The project teams identified two overlapping themes in building climate and health adaptation capacity: professional climate and health equity literacy and collaboration with partners. Building capacity for climate adaptation is uniquely challenging given the scope, complexity, and uncertainty of the impacts and the varying vulnerabilities among different populations. However, through internal and external capacity building guided by the enablers identified here, health departments can better anticipate and overcome barriers in climate and health adaptation. Practitioners can use frameworks such as BRACE that facilitate capacity-building using the principles of adaptive management [5,30,31]. This sequential framework helps health agencies connect anticipated impacts to adaptation planning and implementation and iterative program improvement through monitoring and evaluation.

We identified three primary takeaways to better develop these conditions. First, these projects would not have been possible without dedicated BRACE funding, indicating that dedicated funding or budgeting for climate and health adaptation work is a necessary condition for success. Second, building capacity through institutional knowledge, enhancing climate literacy among staff, and leveraging prior and ongoing work are necessary components to enable successful future work. Researchers and practitioners must continue to highlight effective strategies in the field so new locations may replicate their work or anticipate potential barriers. Third, practitioners can leverage policy windows when problem, policy, and political streams converge. To better anticipate and overcome disruptions, practitioners can learn from the challenges and barriers of peers to enact proactive policies, programs, and interventions that avoid a purely reactive, and thereby disruptive, approach to climate and health adaptation, and by documenting community priorities.

While the work detailed here was scoped to a particular subset of shared environmental hazards, it does not represent all the climate adaptation work among CRSCI grant recipients or elsewhere in the USA. In addition, we are unable to quantifiably demonstrate the effectiveness of the initiatives undertaken until each project can be fully implemented and the associated health outcomes evaluated. Rather, determinations of success were based on the in-context judgment of project teams. Though evaluation is an explicit step in the BRACE framework, recipients were generally unable to conduct rigorous outcome evaluations due to lack of evaluation expertise, resources, and time. The current BRACE funding cycle (CDC-RFA-EH21-2101) places a greater emphasis on both process and outcome evaluation in terms of core activities. Future work is needed to identify successful projects in different locations facing different challenges and in different policy and climate contexts. Further analysis of unfunded jurisdictions and unsuccessful projects is needed for a complete picture of barriers and enablers in climate adaptation.

## 4. Conclusions

This work represents eleven successful climate and health adaptation projects across four jurisdictions in the CDC Climate-Ready States and Cities Initiative and represents the first study of its kind among CRSCI grant recipients. In discussion with team leads and supporting staff, we add evidence to a growing body of literature by identifying the barriers to climate and health adaptation practice resulting from disruptions related to concurrent public health and environmental crises further stressing already limited staff time and resources. We also identified enabling factors that facilitated successful climate and health adaptation and allowed practitioners to overcome these barriers and enhance resilience to climate change. By institutionalizing such enablers, state and local health departments can build capacity to respond to such disruptions or barriers as they encounter them. This work can help other adaptation and health practitioners set up their own projects for success in early stages, allowing for a more efficient and effective response.

As climate change continues to produce increasingly frequent and intense environmental hazards that disproportionately impact populations already affected by economic, environmental, and health inequities, practitioners and policy makers can learn from the successes of the CRSCI project teams to set the necessary underlying conditions to enable successful climate and health adaptation. Iterative evaluation of this and similar work through frameworks such as BRACE, with frequent stakeholder engagement to better understand local population context and needs, can help practitioners anticipate and overcome uncertainties and barriers identified in the literature for continual program improvement. Lessons learned from this work will also be applied to continued CRSCI adaptation work by the four grantees, which are funded from 2021 to 2026 under the cooperative agreement CDC- RFA-EH21-2101 (Building Resilience Against Climate Effects: Implementing and Evaluating Adaptation Strategies that Protect and Promote Human Health). Specifically, lessons learned for addressing simultaneous hazards such as COVID-19 and heat as well as other future unexpected complex hazards are expected to be a point of interest to address in ongoing adaptation efforts.

## Figures and Tables

**Table 1:** Eleven projects implemented by CRSCI grantees determined to be successful in achieving objectives in climate and health adaptation.

Project	Hazards Addressed	Strategy	Intended Outcomes	Partners
California Department of Public Health				
Climate Change and Health Vulnerability Indicators for California (CCHVIs) and Climate Change and Health Vulnerability Indicators Visualization (CCHVIz)	All hazards with prioritization of wildfire, sea level rise, drought, heat, and air pollution.	Compile and publish vulnerability indicator data at smallest spatial scale available with narratives detailing climate impacts by indicator, population, and location.	Improved access to research, data, and maps to be used in vulnerability assessments and planning to direct adaptation resources to communities with disproportionate susceptibility to and risk of climate hazards.	Local health departments, California Natural Resources Agency, California Energy Commission, California Air Resources Board, California Department of Forestry and Fire Protection (CAL FIRE), Governor’s Office of Planning and Research, California Department of Transportation, community-based organizations, consultants, tribes, Asian Pacific Environmental Network (APEN), Strategic Growth Council, California Air Pollution Control Officers, regional and local transportation agencies, research and adaptation subcommittees of California’s interagency Climate Action Team, and California Fire Safe Council.
CalBRACE Adaptation Toolkit	All hazards with prioritization of wildfire, sea level rise, drought, heat, and air pollution.	Develop an online toolkit for public health officials to find evidence-based resources and tools specific to California arranged by BRACE steps.	Improved access for planners to planning tools and guidance, research, data, and maps to align state and local climate and health work with the BRACE framework.	Local health departments, state agencies, community-based organizations, consultants, tribes, and state and regional collaboratives, workgroups, and others applying public health to climate and adaptation planning.
Arizona Department of Health Services				
State-wide School Policies for Heat	Heat	Survey schools to develop recommendations, thresholds, and best practices for heat illness risk reduction for school policies based on statewide review of heat policies.	Reduced heat-related illness among school-age populations statewide.	Department of Education, local University, school district officials, Arizona School Facilities Board Arizona school districts.
Heat Illness Public Awareness	Heat	Publish social media messages to help educate and inform residents about heat illness, prevention measures, and resources available.	Increased awareness of heat safety measures to take during extreme heat days among vulnerable populations.	National Weather Service, academic partners, heat workgroup, and media.
Heat Alerts for the Public	Heat	Awareness of prevention, recognition, and treatment strategies for heat-related illness using the CDC’s stay cool, stay hydrated, and stay informed messaging strategy as well as including the time and scale of National Weather Service Heat Warning and publishing combined information to a Gov Delivery listserv. Messages are given before heat season and during heat season as reminders to prevent future heat illness cases.	Increased number of residents taking safety measures during extreme heat and reduced heat illness.	NWS, school districts, general public, county health departments, emergency management, and hospitals.
Improvement of Cooling Center Management	Heat	Survey vulnerable populations and cooling facility managers to determine gaps in service needs and necessary resources to improve daily operations.	Reduced heat illness cases, higher accessibility to cooling centers among vulnerable populations, decreased barriers to cooling center access (transportation, hours of operation, or other restrictions), increased resources for cooling centers.	Non-profits, county health department, local universities, Area Agency on Aging, and cooling centers.
Oregon Health Authority				
Interagency Climate Equity Workgroup	All hazards	Provide climate equity training to cross-sector partners and develop an interagency Climate Equity blueprint for statewide climate adaptation planning and action.	Increased integration and implementation of Climate Equity best practices in state government climate programs, resulting in increased diversity and inclusion of community leaders in decision making and increased number of community-driven investments to protect the health of the most vulnerable populations.	State agencies (including environmental quality, land use planning, transportation, water resources, housing, and forestry agencies), climate equity consultants, and local climate justice leaders.
Community Listening Sessions	All hazards	Organize listening sessions with diverse communities to discuss connections between social determinants of health, cultural traditions, social capital, and climate resilience.	Increased inclusion of community priorities in climate and health assessment and planning so that government investments reflect the needs and solutions identified by communities most affected by climate change.	Community Health Workers Association, community health workers, community-based organizations, general public, and academic partners.
San Francisco Department of Public Health				
San Francisco Climate Change Coordination Committee	All hazards	Establish and facilitate a work group to coordinate and promote San Francisco Department of Public Health engagement on public health and citywide climate adaptation and mitigation activities.	Increased capacity as a health department to engage in citywide climate adaptation and mitigation efforts, communicate the health role in climate preparedness, and identify gaps and prepare for future climate hazards.	Multiple San Francisco Population Health Division programs, including Population Health, Environmental Health, Office of Policy and Planning, Hospitals, Facilities, Public Health Emergency Preparedness and Response, Community Health Equity and Promotion, Emergency Medical Services, Environmental Justice, Epidemiology and Applied Research, Office of Equity.
Wildfire and Extreme Heat Hospital Toolkit	Extreme heat and air quality	Assess the impact of wildfire smoke and extreme heat on San Francisco’s healthcare facilities via a checklist of strategies to prepare facilities, services, and staff for extreme heat and wildfire smoke events.	Increased awareness of best practices for hospital clinicians, facilities management, and emergency preparedness coordinators to prepare for extreme heat and wildfire smoke events and increased ability to advocate to management about the need to invest in resilient infrastructure.	Hospital clinical staff, emergency preparedness coordinators, and facilities management for all San Francisco hospitals.
Hazard and Climate Resilience Plan	All hazards	Implement Technical Working Group to insert climate and health considerations into the Hazard and Climate Resilience Plan, the San Francisco FEMA-mandated Hazard Mitigation Plan.	Enhanced focus on health in the city-wide climate adaptation framework through both the strategies proposed and the metrics used to evaluate and prioritize the strategies.	All city departments

**Table 2:** Barriers and enablers encountered in each project.

	Barriers					Enablers			
	Competing Demands	Diverse Stakeholder Needs	Staff and Leadership Turnover	Uncertainty and Complexity	Inadequate Resources	Partnerships	Leadership Buy-In	Dedicated Skilled Internal Staff	Policy Window
**California Department of Public Health**									
Climate Change and Health Vulnerability Indicators (CCHVIs) and Climate Change and Health Vulnerability Indicators Visualization (CCHVIz)	X	X	X	X	X	X	X	X	X
CalBRACE Adaptation Toolkit	X	X	X	X	X	X	X	X	X
**Arizona Department of Health Services**									
School Heat Policy	X	X	X	X		X	X	X	
Heat Illness Awareness and Adaptation Strategies	X					X	X	X	X
Heat Alerts	X	X	X			X	X	X	
Cooling Center Evaluation	X		X	X	X	X	X	X	
**Oregon Health Authority**									
Interagency Climate Equity Workgroup		X				X	X	X	X
Community Listening Sessions	X	X	X	X	X	X	X	X	X
**San Francisco Department of Public Health**									
Climate Change Coordination Committee	X	X	X		X	X	X	X	
Hospital Toolkit	X	X		X		X	X	X	
Hazard and Climate Resilience Plan	X	X		X		X	X	X	
